# 390. Can Testing the Environment for SARS-CoV-2 Be a Signal for Staff Infections in Nursing Homes (NHs)?

**DOI:** 10.1093/ofid/ofab466.591

**Published:** 2021-12-04

**Authors:** Gabrielle Gussin, Raveena Singh, Izabela Coimbra Ibraim, Raheeb Saavedra, Thomas Tjoa, Micaila Curtis, Ilhem Messaoudi, Susan S Huang

**Affiliations:** 1 University of California, Irvine; 2 Univeristy of California, Irvine, Irvine, California

## Abstract

**Background:**

Federal mandate requires NHs to perform weekly COVID-19 testing of staff. Testing is effective due to barriers to disclosing mild illness, but it is unclear how long the mandate will last. We explored if environmental samples can be used to signal staff COVID-19 cases as an alternative screening tool in NHs.

**Methods:**

We conducted a cross sectional study to assess the value of environmental sampling as a trigger for COVID-19 testing of NH staff using data from currently performed weekly staff sweeps. We performed 35 sampling sweeps across 21 NHs from 6/2020-2/2021. For each sweep, we sampled up to 24 high touch objects in NH breakrooms (N=226), entryways (N=216), and nursing stations (N=194) assuming that positive samples were due to contamination from infected staff. Total staff and positive staff counts were tallied for the staff testing sweeps performed the week of and week prior to environmental sampling. Object samples were processed for SARS-CoV-2 using PCR (StepOnePlus) with a 1 copy/mL limit of detection. We evaluated concordance between object and staff positivity using Cohen’s kappa and calculated the positive and negative predictive value (PPV, NPV) of environmental sweeps for staff positivity, including the attributable capture of positive staff. We tested the association between the proportion of staff positivity and object contamination by room type in a linear regression model when clustering by NH.

**Results:**

Among 35 environmental sweeps, 49% had SARS-CoV-2 positive objects and 69% had positive staff in the same or prior week. Mean positivity was 16% (range 0-83%) among objects and 4% (range 0-22%) among staff. Overall, NPV was 61% and Cohen’s kappa was 0.60. PPV of object sampling as an indicator of positive staff was 100% for every room type, with an attributable capture of positive staff of 76%, with values varying by room type (Table). Breakroom samples were the strongest indicator of any staff cases. Each percent increase in object positivity was associated with an increase in staff positivity in entryways (7.2% increased staff positivity, P=0.01) and nursing stations (5.7% increased staff positivity, P=0.05).

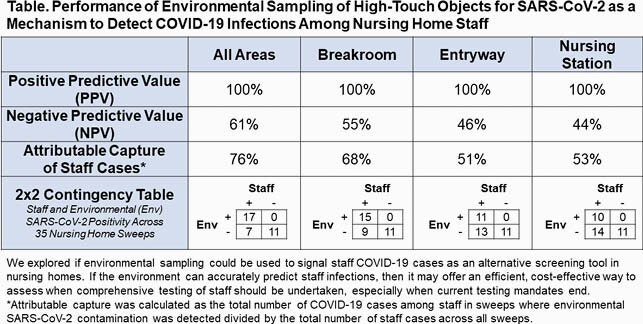

**Conclusion:**

If mandatory weekly staff testing ends in NHs, environmental sampling may serve as an effective tool to trigger targeted COVID-19 testing sweeps of NH staff.

**Disclosures:**

**Gabrielle Gussin, MS**, **Medline** (Other Financial or Material Support, Conducted studies in which participating hospitals and nursing homes received contributed antiseptic and cleaning products)**Stryker (Sage**) (Other Financial or Material Support, Conducted studies in which participating hospitals and nursing homes received contributed antiseptic products)**Xttrium** (Other Financial or Material Support, Conducted studies in which participating hospitals and nursing homes received contributed antiseptic products) **Raveena Singh, MA**, **Medline** (Other Financial or Material Support, Conducted studies in which participating hospitals and nursing homes received contributed antiseptic and cleaning products)**Stryker (Sage**) (Other Financial or Material Support, Conducted studies in which participating hospitals and nursing homes received contributed antiseptic products)**Xttrium** (Other Financial or Material Support, Conducted studies in which participating hospitals and nursing homes received contributed antiseptic products) **Raheeb Saavedra, AS**, **Medline** (Other Financial or Material Support, Conducted studies in which participating hospitals and nursing homes received contributed antiseptic and cleaning products)**Stryker (Sage**) (Other Financial or Material Support, Conducted studies in which participating hospitals and nursing homes received contributed antiseptic products)**Xttrium** (Other Financial or Material Support, Conducted studies in which participating hospitals and nursing homes received contributed antiseptic products) **Susan S. Huang, MD, MPH**, **Medline** (Other Financial or Material Support, Conducted studies in which participating hospitals and nursing homes received contributed antiseptic and cleaning products)**Molnlycke** (Other Financial or Material Support, Conducted studies in which participating hospitals and nursing homes received contributed antiseptic and cleaning products)**Stryker (Sage**) (Other Financial or Material Support, Conducted studies in which participating hospitals and nursing homes received contributed antiseptic and cleaning products)**Xttrium** (Other Financial or Material Support, Conducted studies in which participating hospitals and nursing homes received contributed antiseptic and cleaning products)

